# A preclinical rat model for bilateral phrenic nerve stimulation during mechanical ventilation

**DOI:** 10.1002/ame2.70168

**Published:** 2026-02-27

**Authors:** Jingyi Li, Mulin Zhang, Meizhizi Zhang, Fenqin Xue, Zhize Gao, Xiang Qi, Yongxing Sun, Zhonghua Shi

**Affiliations:** ^1^ Department of Neurosurgery, Intensive Care Unit Sanbo Brain Hospital, Capital Medical University Beijing China; ^2^ Laboratory for Clinical Medicine Capital Medical University Beijing China; ^3^ Laboratory of In Vivo Electrophysiology Core Facility Center of Capital Medical University Beijing China; ^4^ Department of Anesthesiology Sanbo Brain Hospital, Capital Medical University Beijing China; ^5^ Department of Physiology Amsterdam University Medical Centre Amsterdam The Netherlands

**Keywords:** diaphragm electromyography, mechanical ventilation, omohyoid‐based surgery, phrenic nerve stimulation, rat model

## Abstract

Phrenic nerve stimulation (PNS) may preserve diaphragm activation and mitigate multiorgan injury during mechanical ventilation (MV); however, a minimal invasive rat model integrating PNS with MV is lacking. We established an omohyoid muscle‐based PNS rat model combined with MV. Bilateral nerves were exposed within 20 ± 2 min by transection at the intermediate tendon of omohyoid muscle, minimizing trauma and bleeding. Threshold stimulation (0.6 ± 0.2 mA) correlated with body weight. Ventilator‐synchronized stimulation increased compound muscle action potentials by ~30%, whereas histology confirmed intact nerve. Physiological parameters remained stable throughout ventilation. This model provides a safe and scalable platform for mechanistic and preclinical studies on PNS‐mediated protection against MV‐induced organ injury.

## Graphical abstract

In this study, we established a reproducible rat model for bilateral PNS under MV, aiming for a platform for investigating mechanisms and interventions for ventilator associated injury. This model integrating minimal invasive omohyoid‐based surgery, threshold stimulation, and ventilator‐synchronized delivery, demonstrating a stable diaphragm stimulation without detectable phrenic nerve injury.

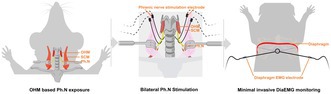

## 1 INTRODUCTION

Although life‐saving, mechanical ventilation (MV) is associated with several complications, including lung injury,^1^ diaphragm dysfunction,^2^ and brain injury,^3^ all of which compromise weaning success and increase mortality in critically ill patients.^3^, ^4^, ^5^ Phrenic nerve stimulation (PNS) has therefore been explored as a strategy to activate the diaphragm during MV, thereby preventing diaphragm atrophy, preserving lung volume and ventilation homogeneity, and attenuating neuroinflammation,^6^ with prior clinical trials and preclinical studies supporting its physiological and therapeutic benefits.^7^, ^8^

Animal models provide a valuable platform for dissecting dose–response relationships and mechanistic pathways that are not feasible in human. Several rat models of PNS have been described, most commonly using sternocleidomastoid muscle (SCM)‐based surgical exposure.^9^, ^10^ Although effective for acute stimulation, this approach is associated with relatively greater muscle disruption and, thus, larger amount of bleeding. Moreover, few studies have integrated invasive PNS with sustained MV in rats, restricting their utility for investigating ventilator‐related multiorgan interactions.

Therefore, we described an omohyoid‐based bilateral cervical approach that minimizes muscle injury and bleeding with sufficient nerve length for electrode placement. We demonstrate the feasibility of stable bilateral PNS during MV diaphragmatic electromyography (EMG) and electrocardiography (ECG) monitoring. This model is intended as a technical and physiological platform for mechanistic studies of PNS on MV‐induced multiorgan injury.

## 2 METHODS

### 2.1 Animals

Male Sprague–Dawley rats (8–12 weeks; Charles River) were housed under specific pathogen‐free conditions (24 ± 2°C, 50% ± 10% humidity, 12‐h light/dark cycle) with food and water ad libitum for 5–7 days before the experiment. All procedures were approved by the Animal Experiments and Experimental Animal Welfare Committee of Capital Medical University (no.: AEEI‐2024‐391).

### 2.2 Preparations

All instruments and the operating table were disinfected with 75% ethanol. Appropriate lighting and a 37°C heating pad were prepared. The anesthesia system (ZS‐MV‐I, Zhongshi Science & Technology) was checked to ensure adequate isoflurane and oxygen supply before connection to the breathing circuit and operating table.

**FIGURE 1:**
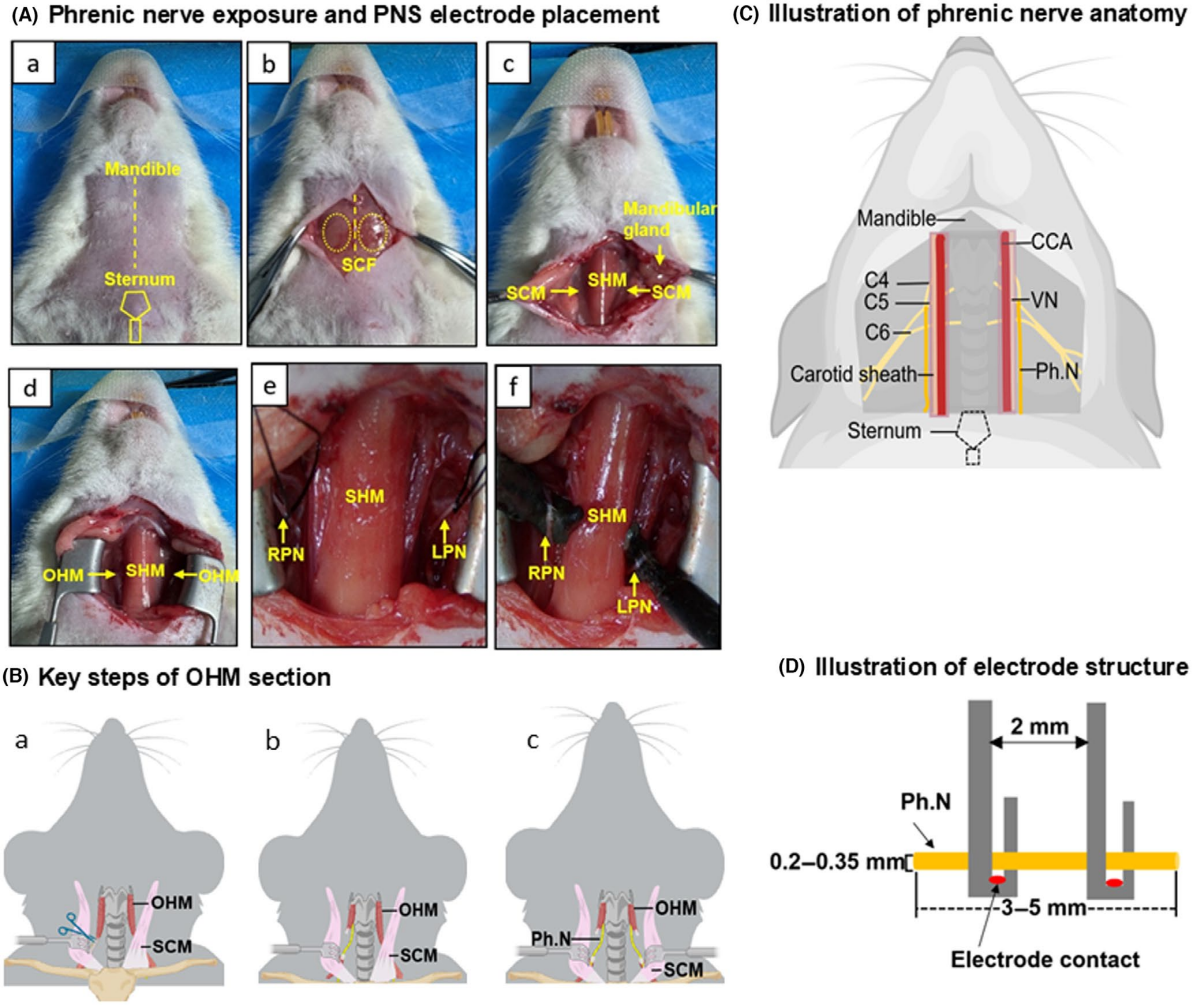
Omohyoid‐based surgical procedures. (A) Phrenic nerve exposure and phrenic nerve stimulation (PNS) electrode placement. (a) Ventral view of the neck showing surgical landmarks. (b) Exposure of superficial cervical fascia. (c) Identification of cervical anatomical landmarks, including mandibular gland, SHM, and SCM. (d) Exposure of OHM with stabilization of SCM. (e) Cervical segments of RPN and left LPN. (f) Implantation of PNS electrodes. (B) Key steps of OHM section. (a) Retract the SCM. (b) Transect OHM. (c) Expose bilateral Ph.N. (C) Illustration of phrenic nerve anatomy. Anatomical location of cervical phrenic nerves. (D) Illustration of electrode structure. Double‐hooked PNS electrode (2 mm width) with two contacts, insulated except at the contact points. CCA, common carotid artery; LPN, left phrenic nerve; OHM, omohyoid muscle; Ph.N, phrenic nerve; RPN, right phrenic nerve; SCF, superficial cervical fascia; SCM, sternocleidomastoid muscle; SHM, sternohyoid muscle; VN, vagus nerve.

### 2.3 Anesthesia and mechanical ventilation

Anesthesia was induced with 3%–4% isoflurane until reflexes disappeared. Rats were positioned supine, and tail vein catheterization was performed under 1.5%–2% isoflurane followed by maintenance anesthesia with intravenous 1% propofol (0.2 mL/100 g bolus, 0.4 mL/100 g/h).

Endotracheal intubation (16‐G catheter) was performed,^11^ which was then connected to a small‐animal ventilator (KW‐100, NJKEWBIO, Nanjing, China) under lung‐protective settings: tidal volume 4–6 mL/kg, positive end‐expiratory (PEEP) 0 cmH_2_O, and respiratory rate 60–80 breath/min with I:E = 1:1.

### 2.4 Phrenic nerves exposure surgery

The ventral neck (lower mandible to upper sternum, ~3–3.5 cm wide) was shaved and disinfected. A midline incision (±1.5 cm) was made followed by blunt dissection. The SCM was retracted to expose the bilateral omohyoid muscles. The omohyoid muscle was transected at its intermediate tendon using fine scissors, creating a clear surgical window with minimal surrounding tissue disruption and negligible bleeding. The phrenic nerve (diameter 0.2–0.35 mm) was visualized along the anterior surface of the scalenus anterior muscle lateral to the common carotid artery and the vagus nerve (0.2–0.5 mm) (enclosed in the carotid sheath). The cervical plexus (C4–C6) contributing to the phrenic nerve was visible. A 3–5 mm segment of phrenic nerve was exposed and secured with 5–0 suture for electrode placement (Figure 1).

### 2.5 Diaphragm EMG and ECG monitoring

**FIGURE 2:**
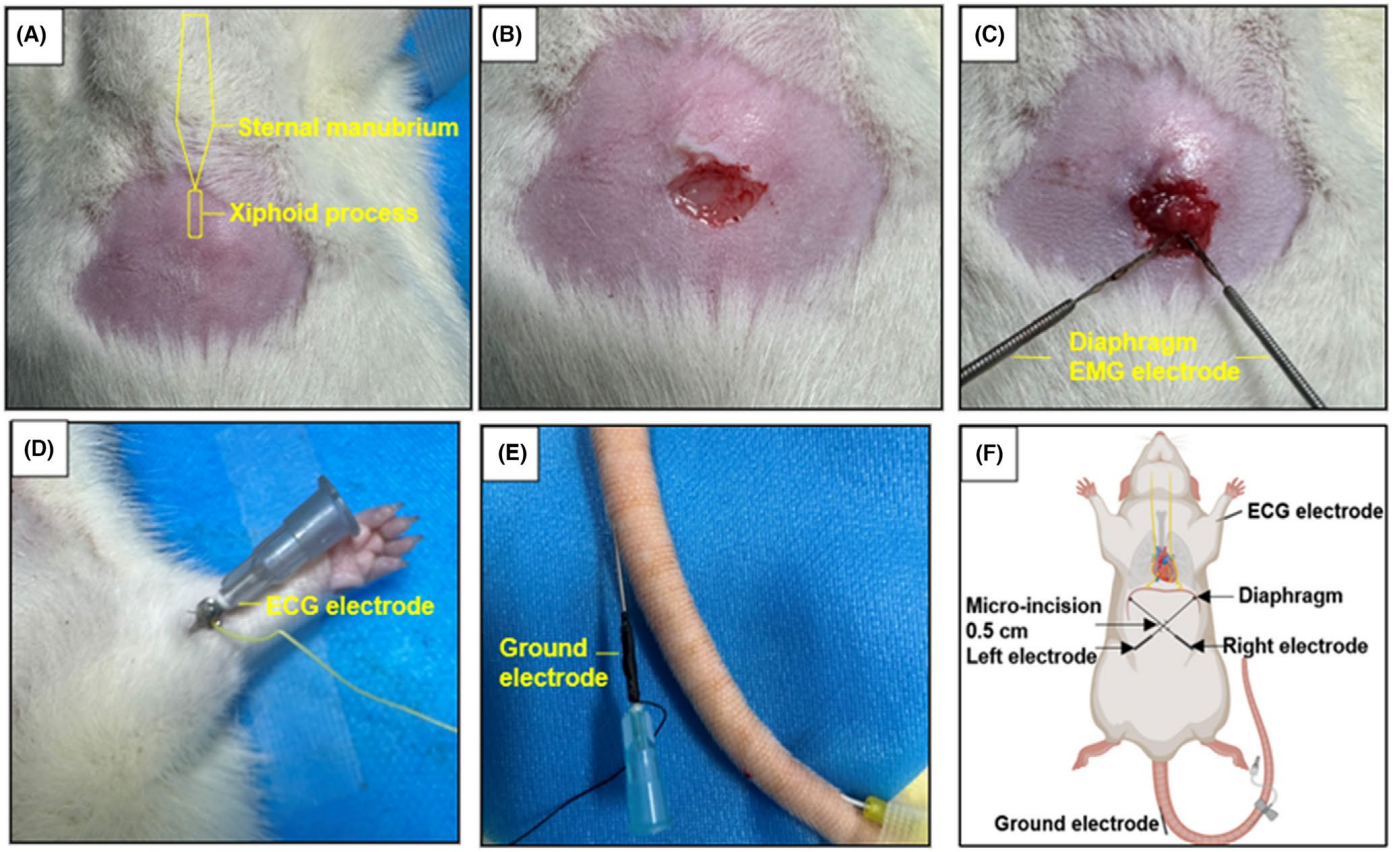
Diaphragm EMG and ECG monitoring. (A) Landmarks of surgical area, showing the sternal manubrium and the xiphoid process. (B) Location of the fistula opening. A 0.5‐cm incision was made beneath the xiphoid process. (C) Implantation of the bilateral diaphragm recording electrodes. (D) Placement of the ECG electrode. (E) Placement of the ground electrode. (F) Schematic diagram of monitoring electrode implantation. ECG, electrocardiogram; EMG, electromyogram.

A small (±0.5 cm) subxiphoid incision was made for bilateral diaphragm EMG electrode insertion cross wisely toward each costophrenic angle. A tail ground electrode was used for EMG, and a positive electrode was placed on the left forelimb for ECG recording, allowing simultaneous recording of diaphragmatic and cardiac activity (Figure 2).

### 2.6 Phrenic nerve stimulation protocol

#### 2.6.1 Intensity stepwise incremental protocol

**FIGURE 3:**
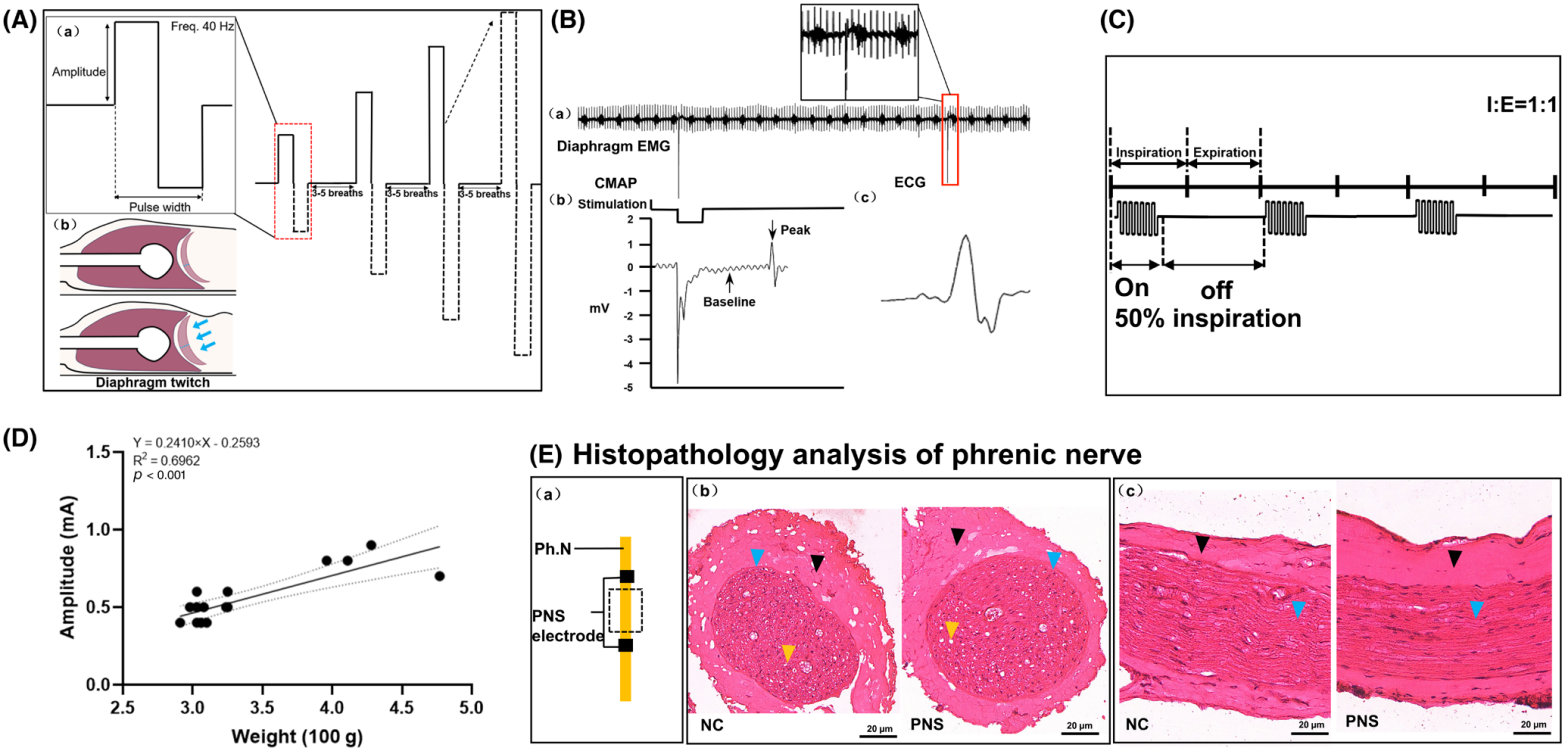
Characterization of phrenic nerve stimulation. (A) Intensity stepwise incremental protocol: PNS amplitude selected using stepwise increment. (B) Electrophysiology: (a) diaphragm EMG during intensity stepwise increment (stimulation every 30 s). (b) CMAP waveform; amplitude measured baseline to peak, showing stimulus artifact and response. (c) ECG waveform. (C) Ventilator‐synchronized PNS. (D) Relationship between CMAP amplitude and rat body weight. (E) Histology (H&E staining) of phrenic nerve: (a) sampling site. (b) cross‐section: NC versus PNS. (c) Longitudinal section: NC versus PNS. Scale bar = 20 μm. Black: epineurium; blue: fascicle; yellow: axon. Nerves preserved with normal axons/myelin, no inflammation or fascicle atrophy. CMAP, compound muscle action potential; ECG, electrocardiogram; EMG, electromyogram; I: E, inspiration: expiration; NC, negative control; PNS, phrenic nerve stimulation.

Handmade bipolar stainless electrodes (double contact points, inter‐contact distance: 2 mm) were connected to a stimulator (Model 2100, A‐M Systems). Biphasic square‐wave pulses (0.1 ms width) were delivered with stepwise current increments (0.1 mA), starting at 0.1 mA, until visible diaphragm contractions of the abdominal wall at the costal margin were observed (Supplemental Video). Contractions were confirmed thrice to ensure reproducibility (Figure 3A). Before and after 6‐h stimulation, compound muscle action potentials (CMAPs) were elicited using the threshold stimulation and recorded from the diaphragm EMG for offline analysis by an independent technician (Figure 3B).

#### 2.6.2 Ventilator‐synchronized stimulation

During controlled MV, stimulation trains (biphasic square‐wave; 0.1 ms pulse width, 40 Hz, with 0.1 mA stepwise current increments) were synchronized to the inspiratory phase (“on” for 50% of inspiration and “off” for the remainder). Stimulation was delivered 10 min per hour over 6 h, with 50 min rest intervals (Figure 3C). Arterial blood gases were measured every 3 h (maintaining PaCO_2_ 25–45 mmHg, PaO_2_ 75–105 mmHg) by adjusting respiratory rate as needed. Anesthesia depth, skin color, ECG, and EMG were continuously monitored.

### 2.7 Postexperiment procedures

After stimulation, rats were euthanized (anesthetic overdose), and tissues were collected per the experimental protocol.

## 3 RESULTS

### 3.1 Surgical procedure

Bilateral cervical phrenic nerves were exposed within 20 ± 2 min using only omohyoid transection, without SCM or sternohyoid muscle resection. This approach minimized bleeding (<2 mL) and tissue trauma. Minor wound exudation (0.1–0.3 mL/h) was managed with sterile moist gauze and balance fluid replacement (0.5–1 mL/100 g/h of a 0.9% NaCl and sodium lactate Ringer's solution).

### 3.2 Stimulation intensity and electrode stability

The stimulation threshold averaged 0.6 ± 0.2 mA and correlated with body weight (intensity = 0.24 × body weight (100 g) − 0.25, *R* ^2^ = 0.70, *p* < 0.001) (Figure 3D). Baseline CMAP amplitude was 0.95 ± 0.25 mV with latency 0.23 ± 0.08 ms; after 6 h stimulation, CMAP amplitude increased to 1.24 ± 0.29 mV (0.29 mV increased compared to baseline, *p* = 0.043) with latency 0.19 ± 0.03 ms, suggesting preserved excitability and neuromuscular stability. Intra‐animal variation decreased from 17.6% ± 8.8% to 12.3% ± 5.8% across repeated measures. Histology (*n* = 6) showed no phrenic injury (Figure 3E). Electrode remained securely in place throughout the 6‐h ventilation without detachment or positional drift.

### 3.3 Ventilator‐synchronized PNS and physiological stability

Ventilator‐synchronized 40‐Hz stimulation was well tolerated, with no asynchrony or abnormal breathing patterns (e.g., tachypnea, bradypnea, irregular chest excursions). Arterial blood gases remained within physiological ranges (PCO_2_ 23–38 mmHg; PO_2_ 75–150 mmHg), and no arrhythmias or hemodynamic instability were observed, confirming physiological stability (Table 1).

**TABLE 1:** Arterial blood analysis and electrolytes during PNS.

Parameter	Baseline	3 h PNS	6 h PNS
pH	7.52 ± 0.09	7.47 ± 0.04	7.53 ± 0.08
PaCO_2_ (mmHg)	28.64 ± 5.91	27.38 ± 11.42	23.02 ± 5.34
PaO_2_ (mmHg)	105.73 ± 28.46	93.89 ± 18.07	121.22 ± 33.28
HCO_3_^−^ (mmol/L)	23.51 ± 5.79	21.81 ± 5.54	19.32 ± 4.41
BE (mmol/L)	0.63 ± 6.73	−1.83 ± 5.83	−3.32 ± 4.99
SaO_2_ (%)	97.33 ± 3.26	97.6 ± 1.04	98.75 ± 1.17
Lac (mmol/L)	1.10 ± 0.25	1.43 ± 0.87	1.51 ± 0.80
Na^+^ (mmol/L)	143.85 ± 7.88	141 ± 4.3	139.33 ± 5.50
K^+^ (mmol/L)	4.9 ± 2.74	4.7 ± 1.30	5.2 ± 1.74
Ca^2+^ (mmol/L)	1.05 ± 0.24	1.12 ± 0.20	1.03 ± 0.21
Cl^−^ (mmol/L)	111.43 ± 10.33	108.71 ± 7.13	111 ± 7.48

*Note*: Data are presented as mean ± standard deviation.

Abbreviations: BE, base excess; PNS, phrenic nerve stimulation.

## 4 DISCUSSION

In this study, we established a reproducible rat model for bilateral PNS under MV, aiming for a platform for investigating mechanisms and interventions for ventilator‐associated injury. This model integrates minimally invasive omohyoid‐based surgery, threshold stimulation, and ventilator‐synchronized delivery, and demonstrates stable diaphragm stimulation without detectable phrenic nerve injury.

Most prior PNS research focuses on large animals, and standardized rat models remain scare. Previous rat models mainly relied on transection of the SCM and sternohyoid muscles to expose the phrenic nerve, resulting in significant surgical trauma and bleeding.^9^ Noninvasive approaches, such as transcutaneous electromagnetic stimulation, provide indirect diaphragm stimulation but lack direct phrenic nerve access.^12^, ^13^ In contrast, our omohyoid‐only approach offers a focused, minimally invasive route for phrenic nerve exposure, allowing electrode placement over 3–5 mm nerve segment. After brief training, the procedure can be completed in approximately 20 min, minimizing delays during the early phase of MV.

Using a stepwise intensity‐increasing protocol, we established a reliable stimulation threshold. A positive correlation between threshold intensity and body weight was observed. This may be influenced by abdominal fat and potentially by nerve diameter, which will be explored in our future studies.

Although PNS is increasingly recognized, precise synchronization with the ventilator's inspiratory phase remains challenging.^14^, ^15^ In our model, aligning stimulation to the first 50% of inspiration minimized ventilator‐stimulation asynchrony and preserved normal breathing patterns. A ~30% increase in CMAP demonstrated improvement of the efficiency of neuromuscular electrical excitation transmission, whereas histology confirmed the absence of phrenic nerve injury under our stimulation strategy. These findings support the safety and efficacy of PNS under controlled mechanical ventilation; however, further studies are needed to optimize synchronization during pressure‐support modes or spontaneous breathing recovery, where respiratory variability may affect stimulation timing.

The integration of diaphragm EMG monitoring though a minimal subxiphoid incision and cross‐wise electrode design accommodated diaphragmatic movement while maintaining stable electrode contact, potentially enabling chronic monitoring through subcutaneous tracts.

This study has several limitations, including the short‐term stimulation, limited histological assessment, and anatomical differences that may limit extrapolation to humans. The dose–response relationship and the functional outcomes were not evaluated and remain subjects of ongoing investigation.

## 5 CONCLUSIONS

We developed a reproducible rat model for bilateral PNS under MV, integrating minimally invasive omohyoid‐based surgery, ventilator‐synchronized stimulation, and real‐time diaphragm monitoring. This platform provides a practical and scalable tool for mechanistic exploration and preclinical evaluation of PNS‐based interventions aimed at preventing MV‐induced diaphragm and brain injury.

## AUTHOR CONTRIBUTIONS

**Jingyi Li:** Methodology; writing – original draft. **Meizhizi Zhang:** Formal analysis; software. **Mulin Zhang:** Formal analysis; software. **Fenqin Xue:** Methodology. **Zhize Gao:** Methodology. **Xiang Qi:** Methodology. **Yongxing Sun:** Writing – original draft. **Zhonghua Shi:** Conceptualization; funding acquisition; supervision; writing – review and editing.

## FUNDING INFORMATION

This work was supported by the Outstanding Young Investigator Program of Capital Medical University (grant no. A2308).

## CONFLICT OF INTEREST STATEMENT

The authors declare no conflicts of interest.

## ETHICS STATEMENT

All procedures were approved by the Animal Experiments and Experimental Animal Welfare Committee of Capital Medical University (no. AEEI‐2024‐391).

## Supporting information


**Video S1.** Diaphragm contraction induced by phrenic nerve stimulation. This video demonstrates the direct response of the diaphragm to phrenic nerve stimulation. Biphasic square‐wave pulses were delivered with increasing current intensity (in 0.1 mA steps, beginning at 0.1 mA) until a visible contraction of the abdominal wall at the costal margin was observed.
