# Supplementary material for: A preclinical rat model for bilateral phrenic nerve stimulation during mechanical ventilation
Source: Animal Model Exp Med. 2026 Feb 27;9(2):416–21. doi: 10.1002/ame2.70168 (PMC13042500; doi:10.1002/ame2.70168)
Supplement: Supplementary file 1 — Video S1. Diaphragm contraction induced by phrenic nerve stimulation. This video demonstrates the direct response of the diaphragm to phrenic nerve stimulation. Biphasic square‐wave pulses were delivered with increasing current intensity (in 0.1 mA steps, beginning at 0.1 mA) until a visible contraction of the abdominal wall at the costal margin was observed. [file AME2-9-416-s001.zip › AME2_70168__author.docx]

**Supplemental Video: Diaphragm contraction induced by phrenic nerve stimulation**

This video demonstrates the direct response of the diaphragm to phrenic nerve stimulation. Biphasic square-wave pulses were delivered with increasing current intensity (in 0.1 mA steps, beginning at 0.1 mA) until a visible contraction of the abdominal wall at the costal margin was observed.
